# Prevalence and effect on survival of pre-treatment sarcopenia in patients with hematological malignancies: a meta-analysis

**DOI:** 10.3389/fonc.2023.1249353

**Published:** 2023-10-06

**Authors:** Jianzhu Xiong, Kangkang Chen, Wen Huang, Mingang Huang, Feiyan Cao, Yiwen Wang, Qifeng Chen

**Affiliations:** ^1^ Department of Public Health, Shaoxing Seventh People's Hospital, Shaoxing, China; ^2^ Department of Non-communicable Diseases Control and Prevention, Shaoxing Center for Disease Control and Prevention, Shaoxing, China; ^3^ Dispatch Division of Shaoxing Emergency Medical Services, Shaoxing Center for Emergency, Shaoxing, China

**Keywords:** hematological malignancies, sarcopenia, prevalence, survival, meta-analysis

## Abstract

**Background & aims:**

Evidence regarding the prevalence of pre-treatment sarcopenia and its impact on survival in patients with hematological malignancies (HM) varies across studies. We conducted a systematic review and meta-analysis to summarize this discrepancy.

**Methods:**

PubMed, Embase and Cochrane library were systematically searched for relevant studies. Outcomes assessed were: prevalence of pre-treatment sarcopenia, overall survival (OS), progression-free survival (PFS) and complete response (CR). Weighted mean proportion, odds ratios (ORs) and hazard ratios (HRs) were estimated using a fixed-effects and a random-effects model.

**Results:**

A total of 27 retrospective cohort studies involving 4,991 patients were included in this study. The prevalence of pre-treatment sarcopenia was 37.0% (95% CI: 32.0%-42.0%) in HM patients <60 years and 51.0% (95% CI: 45.0%-57.0%) in≥60 years. Patients with leukemia had the lowest prevalence, compared with those with other HM (38.0%; 95% CI: 33.0%-43.0%; *P* = 0.010). The presence of sarcopenia was independently associated with poor OS (HR = 1.57, 95% CI = 1.41-1.75) and PFS (HR = 1.50, 95% CI = 1.22-1.83) throughout treatment period, which may be partially attributed to decreased CR (OR = 0.54, 95% CI = 0.41-0.72), particularly for BMI ≥ 25 (*P* = 0.020) and males (*P* = 0.020).

**Conclusion:**

Sarcopenia is highly prevalent in patients with HM and an adverse prognostic factor for both survival and treatment efficacy. HM and sarcopenia can aggravate each other. We suggest that in future clinical work, incorporating sarcopenia into risk scores will contribute to guide patient stratification and therapeutic strategy, particularly for the elderly.

**Systematic review registration:**

https://www.crd.york.ac.uk/prospero/, identifier (CRD42023392550).

## Introduction

Hematological malignancies (HM) represent a mixed group of tumors arising in the blood, bone marrow, lymph, and lymphatic system. These malignancies are classified into four main types: Leukemia, Hodgkin Lymphoma, Non-Hodgkin Lymphoma and Multiple Myeloma. In 2018, HM accounted for approximately 6.6% and 7.2% of all cancer diagnoses and deaths globally, respectively (1). Specifically, the number of new cases and deaths for each HM were as follows: Non-Hodgkin’s Lymphoma (509,590 new cases and 248,724 deaths); Leukemia (437,033 and 309,006); Multiple myeloma (159,985 and 106,105); and Hodgkin Lymphoma (79,990 and 26,167) (1). Of these, more than 60% of HM patients were aged≥60 years and as life expectancy improves, this proportion will increase in the future (2). So far, numerous efforts such as molecular targeted therapy have been developed, leading to great advances observed in almost all HM. However, a substantial proportion of patients fail to achieve disease control. For example, nearly 40% of patients with chronic myeloid leukemia treated with tyrosine kinase inhibitors fail to achieve an optimal response throughout 5-year treatment period, or later relapse (3, 4). Increasing evidence has demonstrated that the prognosis of HM relies not only on HM biology-related factors, such as disease stage, but also on host factors, such as loss of skeletal muscle mass (sarcopenia).

Sarcopenia, caused by aging, disease, inactivity and malnutrition, is a progressive and generalized skeletal muscle disorder (5). It is commonly associated with increased likelihood of poor human health, including physical disability (6), chronic disease states (7, 8), and lowered quality of life (9). Peterson et al. (10) showed that patients with cancer were at an increased risk for sarcopenia, ranging from 15% to 50%. Furthermore, sarcopenia was also shown to have the prognostic value in several cancers, such as lung cancer (11), gastric cancer (12), and esophageal cancer (13).

As two common diseases in the elderly, whether sarcopenia has the predictive value in patients with HM is an active field of current research (14–40). However, the results derived from most of the studies are inconsistent and more than half are even controversial. For example, Besutti et al. (27) showed that sarcopenia did not affect survival in patients with HM. On the other hand, Leone et al. (20) reported that sarcopenia was independently associated with poor survival in patients with HM.

In 2019, Surov et al. (41) conducted a meta-analysis of 7 studies to summarize the impact of sarcopenia on malignant hematological diseases. However, this review assessed the outcome for overall survival only. In addition, as the interest continues to expand in the prognostic value of sarcopenia in patients with HM, the relevant studies have approximately tripled in size from 2019. In view of huge amounts of data obtained from new studies, the sarcopenia prevalence and evidence base of correlation between sarcopenia and HM need to be further updated. Most recently, Xu et al. (42) conducted another meta-analysis to investigate prognostic value of sarcopenia, but the study was restricted to diffuse large B-cell lymphoma. Furthermore, 3 studies included in their meta-analysis of 12 studies were from the same dataset (30, 43, 44), which could lead to biased results. Therefore, we conducted a systematic review and meta-analysis to explore the prevalence of pre-treatment sarcopenia in patients with HM and to ascertain the impact of sarcopenia on clinical outcomes in this population.

## Methods

This meta-analysis was performed according to the PRISMA statement (Preferred Reporting Items for Systematic Reviews and Meta-Analyses) (45). The research protocol was registered and approved in PROSPERO (CRD42023392550).

### Data sources

The electronic databases (PubMed, Embase and Cochrane library) were screened from inception to January 08, 2023 by using the text words (i) “sarcopenia” or “muscle mass” or “muscle index” or “muscle strength” or “muscle quality” or “muscle quantity” or “body composition” and (ii) “lymphoma” or “leukemia” or “myeloma” to identify published studies evaluating the impact of pre-treatment sarcopenia on clinical outcomes in patients with various HM. The detailed search strategies are shown in **Table S1**. Reference lists of included studies were also manually searched to identify any relevant studies that did not come up in the initial search. Only English publications were considered.

### Selection criteria

Studies were included if they met the following criteria: (1) patients: adult patients with HM; (2) exposure: pre-treatment sarcopenia measured by computed tomography (CT) or positron emission tomography/CT; (3) comparison: non-sarcopenia arm; and (4) outcome: prevalence of pre-treatment sarcopenia, overall survival (OS), progression-free survival (PFS) and complete response (CR). Exclusion criteria were as follows: (1) reviews, conference proceedings, short reports, abstracts or case reports; and (2) duplicate studies from the same database (only the most recent study was included in the analysis). Two researchers (Y.W. and M.H.) independently screened the titles and abstracts to evaluate the potential studies. If a study was relevant, the full article was obtained for further reviewed by two independent reviewers (K.C. and F.C.). Any disagreements were resolved in a consensus meeting with a third researcher (Q.C.) as a referee.

### Data extraction and risk of bias assessments

The following data were extracted: lead author, publication year, type of HM, sample size, patient characteristics (including age, sex ratio, BMI and revised international prognostic index), method to measure sarcopenia and their cut-off values, prevalence of sarcopenia, median follow-up, risk of bias, and data on outcomes. The extracted data were checked for accuracy by a third researcher (W.H). Any disagreements were resolved by consensus.

The Newcastle-Ottawa Scale (NOS) for observational studies was used to evaluate the risk of bias of included studies. Two researchers (Y.W. and M.H.) individually evaluated study quality by examining nine items: 1) Representativeness of the sarcopenia cohort, 2) Selection of the non-sarcopenia cohort, 3) Ascertainment of sarcopenia, 4) Demonstration that outcome of interest was not present at start of study, 5) Study controls for age, 6) Study controls for any additional factor, 7) Assessment of outcome, 8) Was follow-up long enough for outcomes to occur, and 9) Adequacy of follow up of cohorts. Each item was scored from 0 to 1, for a total maximum of 9 points. The overall methodological quality of each study was divided into high quality (7-9 points), medium quality (4-6 points) and low quality (≤ 3 points). Any disagreements were resolved in a consensus meeting with a third researcher (W.H.) as a referee.

### Statistical analysis

The required data were extracted from each study. Heterogeneity between summary data was assessed using the *I^2^* statistic. *I^2^* < 50% reflected mild to moderate heterogeneity, and > 50% severe heterogeneity. A random effects model was used to calculate the weighted mean proportion, pooled odds ratios (ORs), hazard ratios (HRs) and 95% confidence intervals (CIs) unless we detected mild to moderate heterogeneity, then a fixed effects model was used. To ascertain robustness of findings, sensitivity analyses were performed by repeating with the random-effect method for mild to moderate heterogeneity, or by removing each study one by one for severe heterogeneity. To identify predictors and explore sources of heterogeneity, exploratory sub-analyses were conducted based on prognostic variables which has been reported in other single studies, including clinical characteristics (HM types, revised international prognostic index, prevalence of sarcopenia, method to measure muscle, SMI, age, sex ratio, BMI and follow-up period) and study characteristics (year of publication and sample size). For variables without appropriate threshold to categorize patients, the medians were calculated according to values reported in each study. Publication bias was estimated using funnel plots and Egger’s regression intercept analysis. Analyses were performed with Stata version 16 (Stata Corp., College Station, TX, USA). All tests were 2-tailed, and *P* < 0.05 was considered statistically significant.

## Results

### Studies retrieved and characteristics

**Figure 1:**
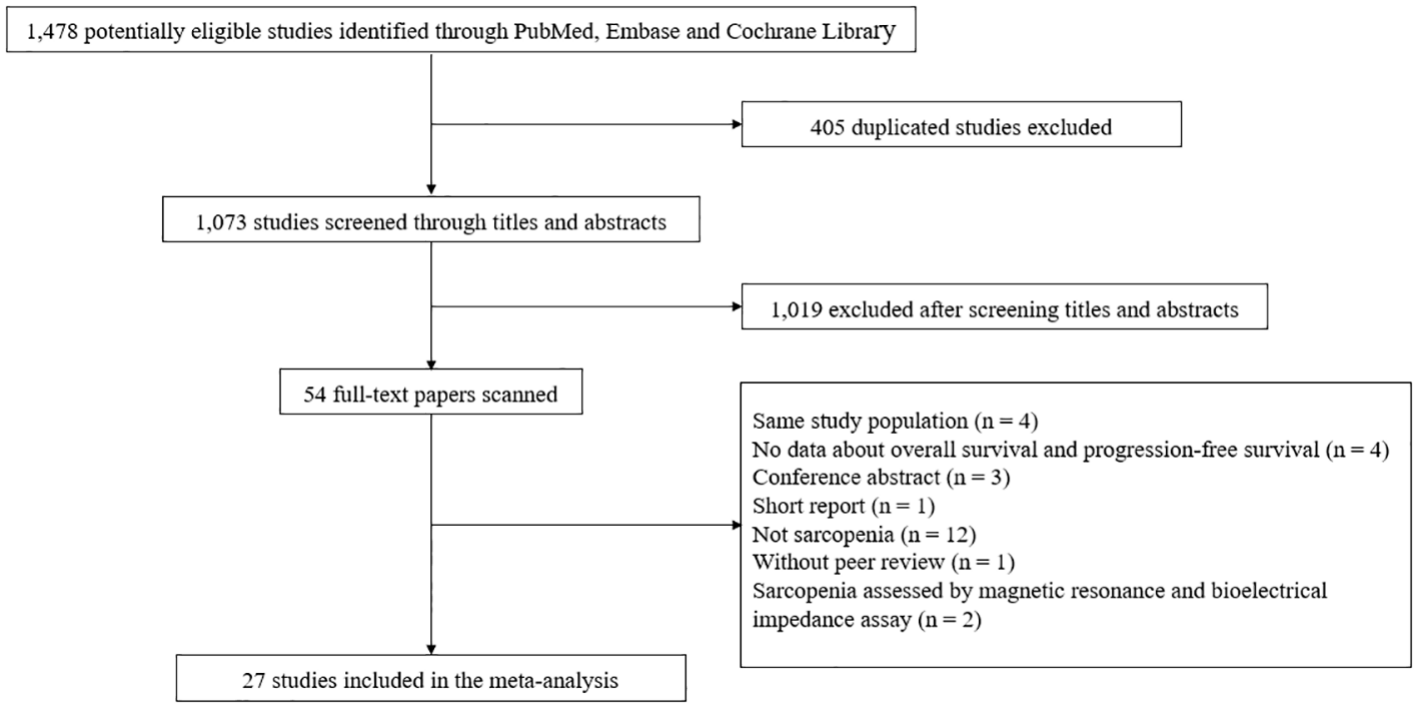
Literature search and screening process.

A total of 1,073 studies were identified after removing duplicates. After screening titles and abstracts, the full text was retrieved for 54 studies. Of these, 27 articles were excluded: 12 did not assess sarcopenia; 4 included the same study population; 4 did not provide HRs for OS and PFS; 3 were conference abstracts; 1 was a short report; 1 has not yet been peer-reviewed; 1 study each used magnetic resonance and bioelectrical impedance assay as a modality to diagnose sarcopenia (**Table S2**). Finally, a total of 27 studies involving 4,991 patients were selected for the final analysis (**Figure 1**).

Among the 27 studies, 18 investigated the impact of sarcopenia in patients with lymphoma (15–18, 20, 23–25, 27–31, 35, 37–40), 4 in patients with leukemia (22, 32–34) and 5 in patients with myeloma (14, 19, 21, 26, 36). Of the 27 CT examinations, the skeletal mass index (SMI) at the third lumbar vertebra level (L3) (CT-L3-SMI) was the most commonly used for the measurement of sarcopenia (14–18, 20, 23, 25–40), followed by the psoas muscle index (PMI) (21, 24), psoas muscle density (PMD) (19) and the SMI at the first lumbar level (L1) (CT-L1-SMI) (22). Of note, although the diagnostic methods to measure sarcopenia were identical across 23 studies, their cut-off values are quite different. The detailed characteristics of the studies and patients are given in **Table 1**. The methodological quality of the included studies was moderate (9 of 27) to high (18 of 27) according to the NOS (**Table S3**). No significant publication bias was observed for OS (*P* = 0.883), PFS (*P* = 0.143) and CR (*P* = 0.346) (**Figure S1**).

**Table 1:** Characteristics of the included trials and participants.

Study	Diagnosis	Total patients	Method to measure sarcopenia	Cut-off value	patients with sarcopenia (%)	Male ratio (%)	Age (median)	BMI (median)	IPI		Median follow-up (months)	Outcome
									0-2 (%)	3-5 (%)		
Lanic 2014	DLBCL	82	CT-L3-SMI	male<55.8cm^2^/m^2^; female<38.9cm^2^/m^2^	54.9	43.9	78.0	24.0	26.8	73.2	39.0	OS, PFS
Camus 2014	DLBCL	80	CT-L3-SMI	male<55.8cm^2^/m^2^; female<38.9cm^2^/m^2^	55.0	43.8	78.7	24.5	27.5	72.5	39.0	OS, PFS
Nakamura 2015	DLBCL	207	CT-L3-SMI	male<47.1cm^2^/m^2^; female<34.4cm^2^/m^2^	56.0	58.5	67.0	NR	53.1	46.9	50.4	OS, PFS
Chu 2015	FL	145	CT-L3-SMI	male<55.8cm^2^/m^2^; female<38.9cm^2^/m^2^	NR	55.0	57.0	27.7	62.8	37.2	NR	OS, CR
Takeoka 2016	MM	56	CT-L3-SMI	male<43cm^2^/m^2^(BMI<25), male<53cm^2^/m^2^(BMI≥25); female<41cm^2^/m^2^	66.0	33.9	71.0	22.1	NR	NR	27.6	OS
Chu 2017	DLBCL	224	CT-L3-SMI	male<53.3cm^2^/m^2^; female<40.2cm^2^/m^2^	NR	56.0	62.0	26.8	49.1	50.9	NR	OS, PFS, CR
Nakamura 2019	AML	90	CT-L3-SMI	male<48.4cm^2^/m^2^; female<33.5cm^2^/m^2^	43.0	56.7	59.0	NR	NR	NR	13.8	OS, PFS, CR
Armenian 2019	AML/ALL/MDS	859	CT-L3-SMI	male<43cm^2^/m^2^(BMI<25), male<53cm^2^/m^2^(BMI≥25); female<41cm^2^/m^2^	33.8	54.0	51.0	NR	NR	NR	NR	OS
Ando 2020	AML/MDS	125	CT-L3-SMI	male<50.9cm^2^/m^2^; female<48.4cm^2^/m^2^	41.6	58.4	44.0	22.4	NR	NR	39.9	OS, PFS
Rier 2020	DLBCL	164	CT-L3-SMI	z-score≤-1	48.8	48.8	64.5	24.8	64.6	35.4	57.0	OS, PFS, CR
Go 2020	DLBCL	228	CT-L3-SMI	male<52.4cm^2^/m^2^; female<38.5cm^2^/m^2^	43.9	57.0	64.0	22.8	55.3	44.7	71.1	OS, PFS
Lin 2020	NHL	146	CT-L3-SMI	male<43cm^2^/m^2^(BMI<25), male<53cm^2^/m^2^(BMI≥25); female<41cm^2^/m^2^	54.8	69.9	60.7	NR	NR	NR	49.4	OS, PFS
Armenian 2020	HL/NHL/DLBCL/MCL/FL	320	CT-L3-SMI	male<43cm^2^/m^2^(BMI<25), male<53cm^2^/m^2^(BMI≥25); female<41cm^2^/m^2^	34.1	61.9	53.3	28.3	NR	NR	NR	OS
Zilioli 2021	HL	154	CT-L3-SMI	male<55cm^2^/m^2^; female<39cm^2^/m^2^	73.0	51.0	71.0	24.2	34.0	66.0	70.8	OS, PFS, CR
da Cunha 2021	MM	91	CT-L3-SMI	male<43cm^2^/m^2^(BMI<25), male<53cm^2^/m^2^(BMI≥25); female<41cm^2^/m^2^	40.7	57.1	64.0	24.8	NR	NR	20.0	OS, PFS
Williams 2021	MM	142	CT-PM-SMD	Psoas density ≤ 80	51.0	65.0	62.4	28.9	NR	NR	30.1	OS, PFS
Jung 2021	AML	96	CT-L1-SMI	male<40.79cm^2^/m^2^; female<31.6cm^2^/m^2^	37.5	52.1	58.0	NR	NR	NR	21.5	OS, PFS, CR
Besutti 2021	DLBCL	116	CT-L3-SMI	male<43cm^2^/m^2^(BMI<25), male<53cm^2^/m^2^(BMI≥25); female<41cm^2^/m^2^	25.0	51.7	63.7	25.4	48.3	51.7	30.0	OS, PFS
Leone 2021	DLBCL	43	CT-L3-SMI	male<41.4cm^2^/m^2^; female<31.0cm^2^/m^2^	30.2	34.9	61.0	25.5	NR	NR	23.0	OS, PFS
Jullien 2021	DLBCL	656	CT-L3-SMI	male<55cm^2^/m^2^; female<39cm^2^/m^2^	34.3	55.9	48.0	24.1	42.5	57.5	36.6	OS, PFS
Guo 2021	DLBCL	201	CT-L3-SMI	≤27.55cm^2^/m^2^	23.9	56.7	56.9	23.1	81.6	18.4	NR	OS, PFS
Iltar 2021	DLBCL	120	CT-PM-SMI	male<440.4mm^2^/m^2^; female<306.87mm^2^/m^2^	54.2	55.0	59.0	26.6	59.2	40.8	NR	OS, PFS, CR
Koyuncu 2021	MM	111	CT-PM-SMI	male<540mm^2^/m^2^; female<360mm^2^/m^2^	41.4	48.7	64.0	26.8	NR	NR	21.7	OS
Albano 2022	MCL	53	CT-L3-SMI	male<53cm^2^/m^2^; female<45.6cm^2^/m^2^	60.0	74.0	72.7	26.2	23.0	77.0	50.0	OS, CR
Albano 2022	HL	88	CT-L3-SMI	male<55cm^2^/m^2^; female<39cm^2^/m^2^	66.0	46.6	72.8	24.9	NR	NR	47.5	OS, PFS, CR
Nandakumar 2022	MM	322	CT-L3-SMI	male<55cm^2^/m^2^; female<39cm^2^/m^2^	53.1	62.0	66.0	26.7	NR	NR	72.0	OS
Ferraro 2022	DLBCL	72	CT-L3-SMI	male<52.4cm^2^/m^2^; female<38.5cm^2^/m^2^	51.4	51.4	68.0	NR	NR	NR	NR	OS, PFS

DLBCL, diffuse large B cell lymphoma; FL, follicular lymphoma; MM, multiple myeloma; AML, acute myeloid leukemia; ALL, acute lymphoid leukemia; MDS, myelodysplastic syndrome; HL, Hodgkin lymphoma; NHL, non-Hodgkin’s lymphoma; MCL, Mantle cell lymphoma; CT, computed tomography; L3, the third lumbar vertebra level; L1, the first lumbar vertebra level; PM, psoas muscle; SMI, skeletal mass index; SMD, skeletal mass density; BMI, body mass index; IPI, international prognostic index; OS, overall survival; PFS, progression-free survival; CR, complete response; NR, not reported.

### Prevalence of sarcopenia prior to treatment

**Figure 2:**
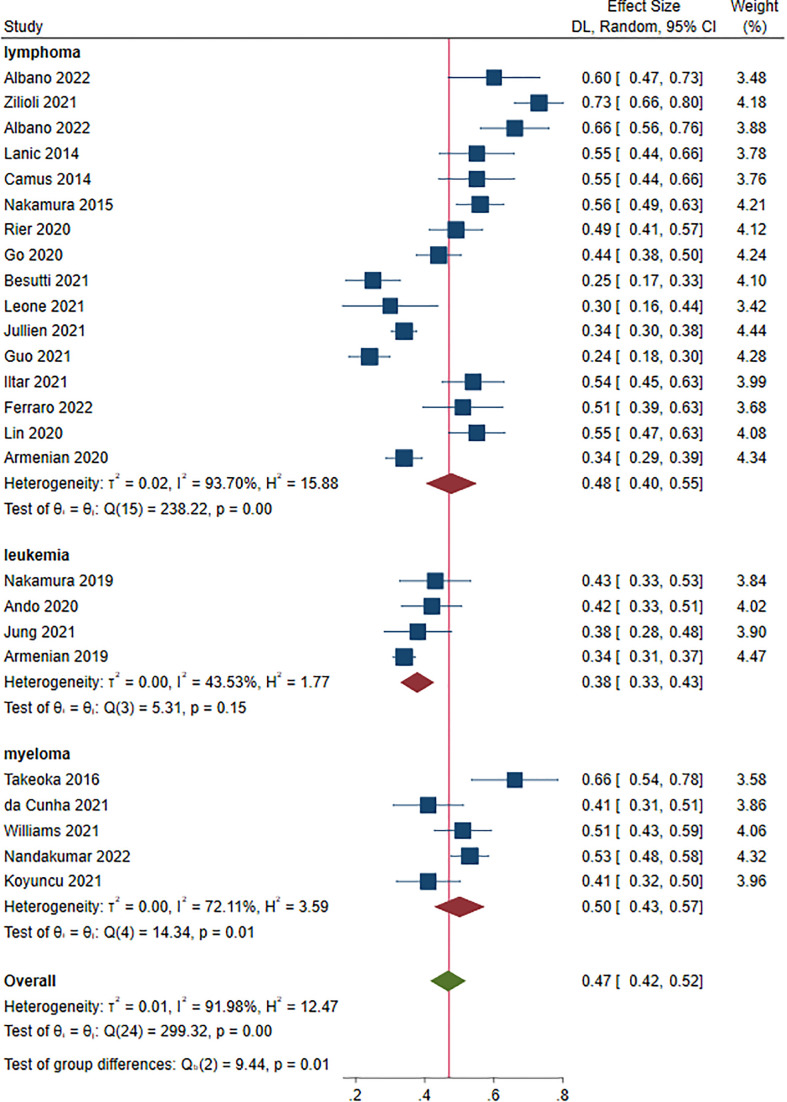
Prevalence of pre-treatment sarcopenia in patients with hematological malignancies.

Twenty-five studies, 4,622 patients, were included in this analysis (14–34, 36, 37, 39, 40). The prevalence of sarcopenia ranged from 23.9% to 73.4% prior to treatment. The overall prevalence of sarcopenia was 47.0% (95% CI: 42.0%-52.0%) at a random-effect model (**Figure 2**). The sensitivity analysis showed that excluding these studies 1 by 1, the overall prevalence did not change (**Figure S2**).

The prevalence for each disease was as follows: lymphoma: 48.0% (95% CI: 40.0%-55.0%, 16 studies) (15–18, 20, 23–25, 27–31, 35, 37–40); leukemia: 38.0% (95% CI: 33.0%-43.0%, 4 studies) (22, 32–34); myeloma: 50.0% (95% CI: 43.0%-57.0%, 5 studies) (14, 19, 21, 26, 36). A significant difference was found in terms of sarcopenia prevalence in patients with different HM (*P* = 0.010)(**Figure 2**).

**Figure 3:**
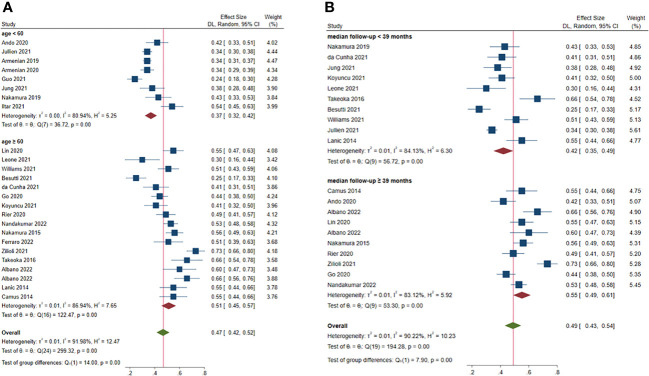
Subgroup analysis of factors contributing to increased prevalence of pre-treatment sarcopenia. **(A)** age; **(B)** median follow-up.

Subgroup analysis was also classified based on the median age of the patients as < 60 and ≥ 60 years. The estimated prevalence of sarcopenia was 37.0% (95% CI: 32.0%-42.0%, 8 studies) (22–25, 31–34) and 51.0% (95% CI: 45.0%-57.0%, 17 studies) (14–21, 26–30, 36, 37, 39, 40) in studies with patient median age < 60 and ≥ 60 years, respectively (*P* < 0.001) (**Figure 3A**).

Median of the mean follow-up of the studies was 39 months. The estimated prevalence of sarcopenia was 41% (95% CI: 34.0%-47.0%, 9 studies) (19–23, 26, 27, 33, 36) and 55% (95% CI: 49.0%-61.0%, 11 studies) (14, 16–18, 28–30, 32, 37, 39, 40) in studies with follow-up < 39 and ≥ 39 months, respectively (*P* < 0.001) (**Figure 3B**).

In addition to types of disease, age and follow-up period, we found no significant differences in prevalence of sarcopenia in subgroup analyses of sex ratio, BMI, revised IPI, method to measure sarcopenia, sample size and publication year (data not shown).

### Overall survival and sarcopenia

**Figure 4:**
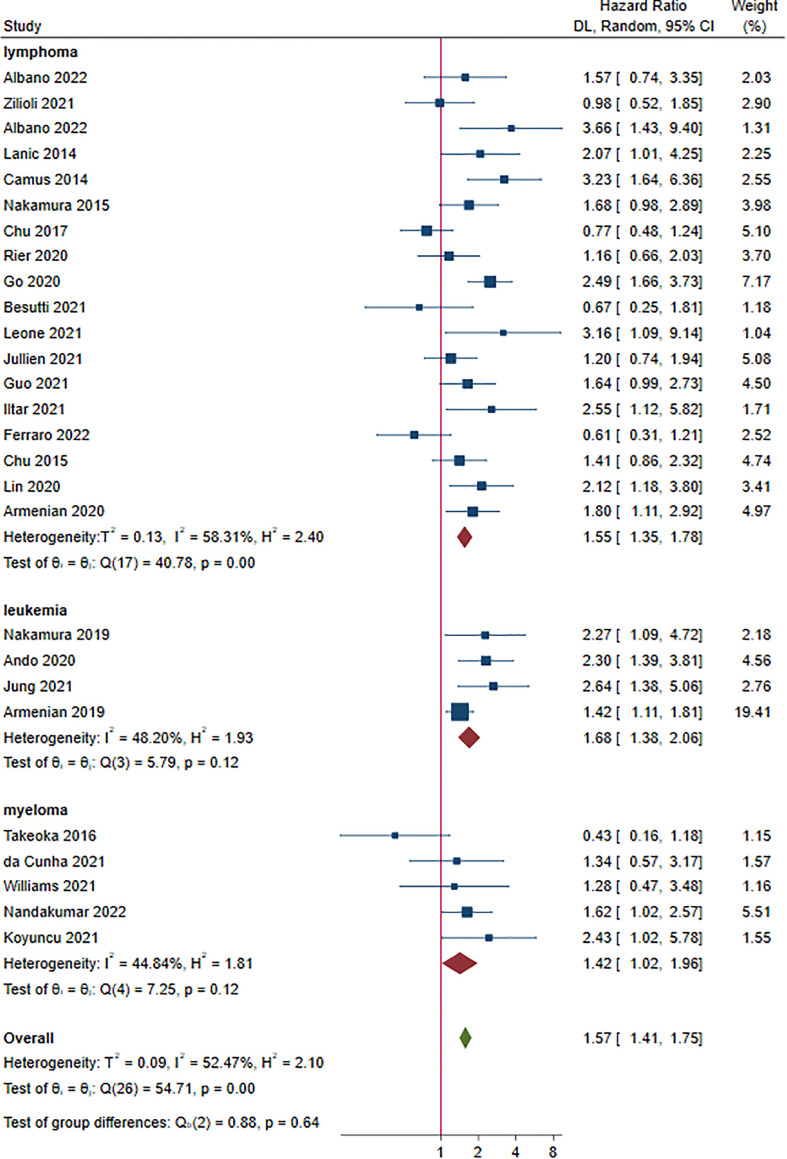
Hazard ratios of sarcopenia for overall survival stratified by cancer type.

Twenty-seven studies reported OS as an outcome (14–40). The HRs for OS ranged from 0.43 to 3.66. Multivariate Cox regression was performed in 17 studies (14, 16, 19, 20, 22, 24, 26–29, 31–33, 36, 37, 39, 40). As shown in **Figure 4**, sarcopenia prior to treatment was shown to have a shorter OS in patients with HM (HR 1.57, 95% CI 1.41-1.75, *I^2^ = *52.47%). Lymphoma, leukemia and myeloma were reported in eighteen (15–18, 20, 23–25, 27–31, 35, 37–40), four (22, 32–34) and five studies (14, 19, 21, 26, 36), respectively. The pooled HRs (95% CIs) were 1.55 (1.35-1.78) for lymphoma, 1.68 (1.38-2.06) for leukemia and 1.42 (1.02-1.96) for myeloma. There were no significant differences among the different malignant hematological diseases (*P* = 0.640). The sensitivity analysis showed that removing studies 1 by 1 did not change the overall results (**Figure S3**).

Subgroup analyses showed that HRs for OS favored non-sarcopenia in almost all variables and no significant difference between HRs was found for each variable (**Table S4**).

### Progression-free survival and sarcopenia

**Figure 5:**
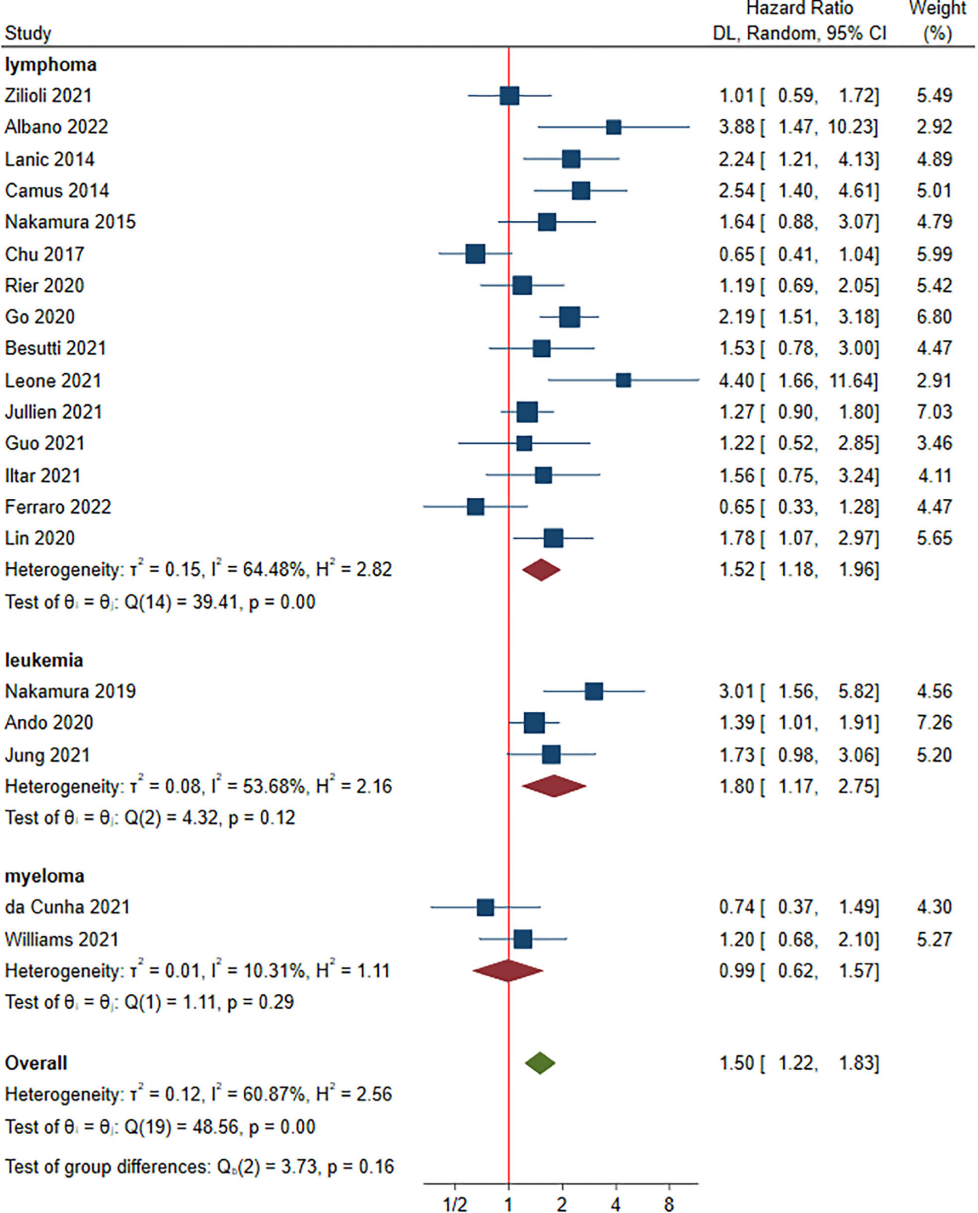
Hazard ratios of sarcopenia for progression-free survival stratified by cancer type.

A total of 20 studies involving 3,125 patients were included in this part (15, 17–20, 22–30, 32, 33, 35, 37, 39, 40). Of these, the HRs for PFS ranged from 0.65 to 4.40. Multivariate analysis was performed in 14 studies (17, 19, 20, 22, 24–29, 32, 33, 37, 39). Compared to non-sarcopenia, the patients with sarcopenia had a negative effect on PFS, with a HR of 1.50 (95% CI: 1.22-1.83, *I^2^ = *60.87%) (**Figure 5**). Lymphoma, leukemia and myeloma were reported in fifteen (15, 17, 18, 20, 23–25, 27–30, 35, 37, 39, 40), four (22, 32, 33) and two studies (19, 26), respectively. The pooled HRs (95% CIs) were 1.52 (1.18-1.96) for lymphoma, 1.80 (1.17-2.75) for leukemia and 0.99 (0.62-1.57) for myeloma. No significant difference in HR was found between different malignant hematological diseases (*P* = 0.160). The sensitivity analysis showed that removing studies 1 by 1 did not change the overall results (**Figure S4**).

Subgroup analyses showed that HRs for PFS favored non-sarcopenia in almost all variables and no significant difference between HRs was found for each variable (**Table S5**).

### Complete response and sarcopenia

**Figure 6:**
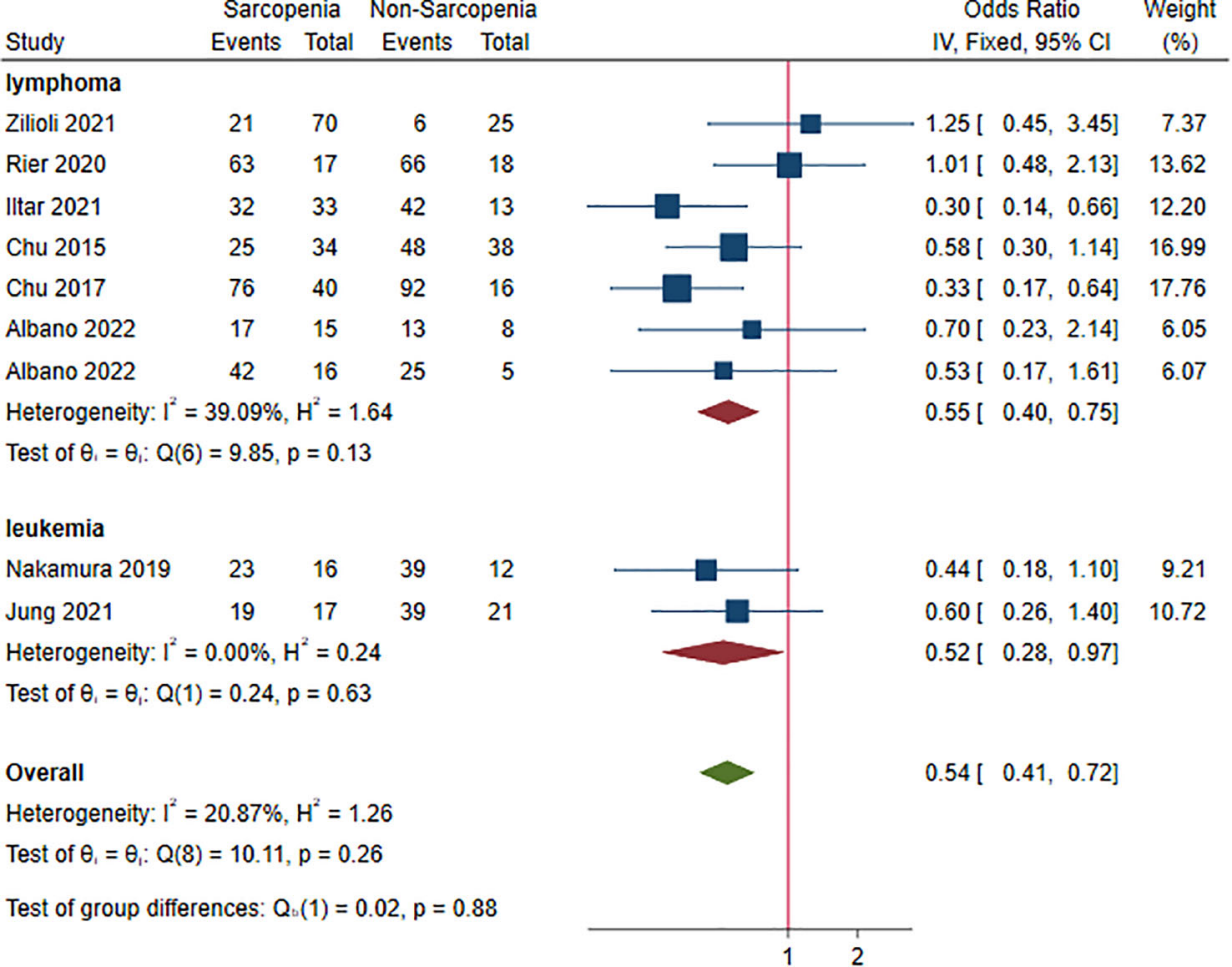
Odds ratios of sarcopenia for complete response stratified by cancer type.

Nine studies, 1,102 patients, were included in this analysis (16–18, 22, 24, 28, 33, 35, 38). The ORs for CR ranged from 0.30 to 1.25. Sarcopenia decreased the rate of CR (OR 0.54, 95% CI 0.41-0.72, *I^2 = ^* 20.87%; **Figure 6**). Lymphoma and leukemia were reported in seven (16–18, 24, 28, 35, 38) and two (22, 33) studies, respectively. The pooled ORs (95% CIs) were 0.55 (0.40-0.75) for lymphoma and 0.52 (0.28-0.97) for leukemia. There was no significant difference between the two HM (*P* = 0.880). In sensitivity analysis, the random-effect method did not change the result, suggesting robustness of analysis to the fixed-effect model (**Figure S5**).

**Table S6** summarizes results of subgroup analysis with respect to potential risk factors for failing to achieve CR. Statistically significant differences were observed within subgroups based on sex ratio (*P* = 0.020) and BMI (*P* = 0.020). Sarcopenia was associated with poor CR in patients being male (OR 0.42, 95% CI 0.30-0.60) and BMI≥25(OR 0.42, 95% CI 0.29-0.62) (**Table S6**).

## Discussion

This meta-analysis of retrospective studies in patients with HM focused on the impact of sarcopenia on survival and efficacy but also on the prevalence of sarcopenia prior to treatment. CT has been accepted as a gold standard to assess muscle quantity/mass and was routinely used before treating HM (46). Considering comparability between the instruments used to measure muscle mass, studies in which sarcopenia was diagnosed by CT only were included in our analysis. The overall prevalence of sarcopenia was 47.0% in patients with HM prior to treatment. Subgroup analyses suggested that advanced age (≥60 years) and long-term follow-up was shown to have a significantly higher prevalence, while a significantly lower prevalence was observed in leukemia. Importantly, the presence of sarcopenia was independently associated with poor OS and PFS throughout treatment period, which can be partially attributed to decreased CR, specific for BMI ≥ 25 and males.

Recently, a meta-analysis conducted by Petermann-Rocha et al. (47) estimated the prevalence of sarcopenia across the globe. Based on 151 studies, the global prevalence of sarcopenia ranged from 10.0% to 27.0% in total population. The result did not change in the elderly as the average age in approximately 92.3% of studies was more than 60 years. Obviously, the sarcopenia prevalence was very high in patients with HM (47.0%) as compared to the general population, and even higher in HM patients older than 60 years (51.0%). The causes of HM-related sarcopenia have not yet been elucidated, and the possible mechanisms are as follows: systemic inflammatory (48), metabolic derangement (49) and mitochondrial function (50). Besides these, limited physical activity and poor nutrition intake can also be observed in patients with HM, resulting in non-cancer biology-related decrease in muscle mass. It has been recognized that aging was a significant risk factor for sarcopenia (51), which was also proved to be applicable to HM patients in our subgroup analysis by age (51.0% vs 37.0%). Thus, our study suggested that people with HM and/or advanced age had more likelihood of sarcopenia. One problem we have not yet solved is the effect size of the pathological condition resulted from HM alone and physiological condition resulted from advanced age alone on sarcopenia. Also, whether HM and aging act synergistically to increase the risk of developing sarcopenia is worthy of being investigated in the future. Our study provided pooled prevalence of sarcopenia which can be regarded as a reference for the calculation of sample size for future intervention studies.

The similar difference between the general and HM populations in terms of increased sarcopenia was also observed in non-hematological solid tumors, such as lung cancer, renal cell carcinoma, hepatocellular carcinoma, melanoma, etc. (11, 52, 53) However, our study found that prevalence of sarcopenia was significantly lower for patients with leukemia. It is possible that the lower prevalence observed could be as a result of young patients, since the median age in all studies included in the leukemia subgroup analysis was less than 60 years compared with other cohorts.

Importantly, our study suggested that sarcopenia may serve as an adverse prognostic factor for both OS and PFS in patients with HM. But the HR for PFS in myeloma was no statistical difference. The result may be due to the limited number of studies included (2 studies comprising only 233 patients). The poor survival profiles in patients with HM and sarcopenia can be explained by the drug-related adverse effects. Previous studies demonstrated that lower muscle mass and the resulting reduction in the clearance of anti-tumor drugs were associated with serious toxicities during or after chemotherapy (54, 55). For example, Guo et al. (25) demonstrated that for every 5 cm^2^/m^2^ decrease in SMI, the risk of any grade 3-4 toxicity was increased by 34%. Nakamura et al. (33) even found that all sarcopenia patients older than 60 years died within 1 year after induction chemotherapy. Besides, dose reductions or interruption of treatment resulted from toxicities of therapy may also add to the poor prognosis for sarcopenic patients (56). Another potential explanation for the favorable survival observed in non-sarcopenia patients was that numerous cytokines (e.g., myokines) released by skeletal muscle cells can inhibit cancer cell viability and proliferation (57).

Although a substantial proportion of studies (7 of 9) denied the role of sarcopenia in terms of CR, the pooled CR was significant. This result is reasonable because the mechanisms mentioned above influencing OS and PFS can also influence the treatment efficacy such as early discontinuation of treatment due to increased toxicities. Interestingly, the high proportion of males and BMI ≥ 25 were linked to poor CR. Currently, BMI is the most common indicator used for diagnosing overweight and obesity. BMI ≥ 25 means that increased adipose tissue may decrease muscle mass, thereby increasing treatment-related toxicities. However, males tend to have a high fat-free mass. It is opposite to the role of BMI on CR, which was described as the ‘obesity paradox’ (58, 59), and needs further investigation.

The overall findings of this study demonstrated that HM and sarcopenia can interact and aggravate each other. Of note, medical treatment can stimulate muscle wasting. For example, Albano et al. (17) found that the rate of sarcopenia patients increased from 66% before chemotherapy to 83% at the end of the treatment; Xiao et al. (60) showed that sarcopenia increased by 26.1% after treatment. Given the wide use of CT scan at the time of diagnosis of HM, we suggested that sarcopenia should be incorporated into the existing prognostic system and given appropriate weight to guide the therapeutic strategy. Also, our findings highlight the importance of treating sarcopenia, in order to minimize adverse consequences. Available evidence suggests that nutrition may improve muscle mass (61), short-chain fatty acids (SCFAs) in particular, which are considered to be involved in the change of muscle biology (62). On the other hand, immune nutrition with some special nutrients has been widely used in patients with cancers, showing different supporting functions (63, 64). Therefore, adding nutrients like SCFAs to the formula of nutrition may help to treat both diseases.

Our meta-analysis has several limitations: 1) The cut-off values of SMI used to assess sarcopenia were not uniform. For example, the standards adopted in some studies were based on values given by Prado et al (65) or Martin et al (66), while values adopted in others were calculated from ROC curve based on their own samples. This may affect the conclusion, as demonstrated by Zilioli et al (18) with different results resulted from the same population. Thus, future studies are warranted to determine optimal cut-off levels when assessing sarcopenia with CT, specific for gender and ethnicity. 2) As the physical performance was not available due to the retrospective nature of the included studies, our meta-analysis cannot investigate the impact of severe sarcopenia on survival and efficacy. 3) Studies were pooled with different patient characteristics such as age, BMI, adipose tissue, etc. Due to this limitation, heterogeneity was severe for the majority of outcomes. We minimized the influence of heterogeneity by subgroup analyses and no significant difference between HRs was found for each variable. Sensitivity analyses were also performed in response to the severe heterogeneity. Both approaches provided concordant results. 4) As with any meta-analysis, our dataset was founded on each included study, and hence several missing variables with the prognostic value such as advanced stage at diagnosis, serum albumin and C-reactive protein may not be evaluated. 5) For some outcomes, the number of studies included was limited, which could increase uncertainty of the results. 6) As this meta-analysis was carried out in patients with HM, our findings cannot be used as a reference for improving the prognosis of non-hematological solid tumors.

## Conclusions

In conclusion, the prevalence of pre-treatment sarcopenia is found to be very high in patients with HM, and even higher in HM patients older than 60 years. The presence of sarcopenia is independently associated with poor survival and treatment response throughout treatment period. Our meta-analysis suggests that HM and sarcopenia can aggravate each other. In future clinical work, sarcopenia screening prior to treating HM will contribute to guide patient stratification and therapeutic strategy, particularly for the elderly.

## Data availability statement

The original contributions presented in the study are included in the article/**Supplementary Material**. Further inquiries can be directed to the corresponding author.

## Author contributions

JX, KC and QC contributed to the conception and design this study. WH and MH carried out the development of the methodology. KC, MH and YW analyzed and interpreted the data. JX, KC and QC wrote the manuscript and approved the final submission of the study. All authors read and approved the final manuscript.
