# Supplementary material for: Prevalence and effect on survival of pre-treatment sarcopenia in patients with hematological malignancies: a meta-analysis
Source: Front Oncol. 2023 Oct 6;13:1249353. doi: 10.3389/fonc.2023.1249353 (PMC10587577; doi:10.3389/fonc.2023.1249353)
Supplement: Supplementary file 1 [file DataSheet_1.docx]

**Supplemental Files**

**Table S1** Search Strategy and Search Results

| **Table S1** Search Strategy and Search Results | | |
| --- | --- | --- |
| **A** PubMed Search | | |
| Entry | PubMed Search Strategy | Results |
| 1 | sarcopenia OR muscle mass OR muscle index OR muscle strength OR muscle quality OR muscle quantity OR body composition | 130,714 |
| 2 | lymphoma OR leukemia OR myeloma | 578,413 |
| 3 | #1 AND #2 | 400 |
| **B** Embase Search | | |
| Entry | Embase Search Strategy | Results |
| 1 | sarcopenia OR muscle mass OR muscle index OR muscle strength OR muscle quality OR muscle quantity OR body composition | 219,308 |
| 2 | lymphoma: ti, ab, kw OR leukemia: ti, ab, kw OR myeloma: ti, ab, kw | 656,961 |
| 3 | #1 AND #2 | 868 |
| **C** Cochrane Library Search | | |
| Entry | Cochrane Library Search Strategy | Results |
| 1 | sarcopenia OR muscle mass OR muscle index OR muscle strength OR muscle quality OR muscle quantity OR body composition | 58,448 |
| 2 | (b3a2) OR (b2a2) OR (e14a2) OR (e13a2) | 30,838 |
| 3 | #1 AND #2 | 210 |

**Table S2** The reasons for the exclusion of full-text articles

| Same study population (n=4)^1-4^ |
| --- |
| No data about overall survival and progression-free survival (n = 4)^5-8^ |
| Conference abstract (n = 3)^9-11^ |
| Not sarcopenia (n = 12)^12-23^ |
| Without peer review (n = 1)^24^ |
| Short report (n = 1)^25^ |
| Sarcopenia assessed by magnetic resonance and bioelectrical impedance assay^26,27^ |

**Table S3**. Newcastle-Ottawa Scale for assessing the quality of comparative studies in the meta-analysis

| **Study** | **Representativeness of the sarcopenia cohort^*^** | **Selection of the non- sarcopenia cohort** | **Ascertainment of sarcopenia** | **Demonstration that outcome of interest was not present at start of study** | **Study controls for**  **age** | **Study controls for any additional factor** | **Assessment of outcome** | **Was follow-up long enough for outcomes to occur** | **Adequacy of follow up of cohorts** |
| --- | --- | --- | --- | --- | --- | --- | --- | --- | --- |
| Albano 2022 | - | ★ | ★ | ★ | ★ | ★ | ★ | ★ | ★ |
| Zilioli 2021 | ★ | ★ | ★ | ★ | - | ★ | ★ | ★ | ★ |
| Albano 2022 | **‐** | ★ | ★ | ★ | ★ | ★ | ★ | ★ | ★ |
| Takeoka 2016 | **-** | ★ | ★ | ★ | - | - | ★ | ★ | ★ |
| da Cunha 2021 | ★ | ★ | ★ | ★ | - | ★ | ★ | ★ | ★ |
| Williams 2021 | **-** | ★ | ★ | ★ | ★ | ★ | ★ | ★ | ★ |
| Nandakumar 2022 | - | ★ | ★ | ★ | ★ | ★ | ★ | ★ | ★ |
| Nakamura 2019 | - | ★ | ★ | ★ | ★ | ★ | ★ | ★ | ★ |
| Ando 2020 | ★ | ★ | ★ | ★ | ★ | ★ | ★ | ★ | ★ |
| Jung 2021 | - | ★ | ★ | ★ | ★ | - | ★ | ★ | ★ |
| Koyuncu 2021 | - | ★ | ★ | ★ | - | - | ★ | ★ | ★ |
| Lanic 2014 | - | ★ | ★ | ★ | - | - | ★ | ★ | ★ |
| Camus 2014 | - | ★ | ★ | ★ | - | - | ★ | ★ | ★ |
| Nakamura 2015 | - | ★ | ★ | ★ | - | ★ | ★ | ★ | ★ |
| Chu 2017 | - | ★ | ★ | ★ | - | ★ | ★ | ★ | ★ |
| Rier 2020 | - | ★ | ★ | ★ | ★ | - | ★ | ★ | ★ |
| Go 2020 | - | ★ | ★ | ★ | - | - | ★ | ★ | ★ |
| Besutti 2021 | - | ★ | ★ | ★ | - | - | ★ | ★ | ★ |
| Leone 2021 | - | ★ | ★ | ★ | - | - | ★ | ★ | ★ |
| Jullien 2021 | ★ | ★ | ★ | ★ | - | - | ★ | ★ | ★ |
| Guo 2021 | - | ★ | ★ | ★ | - | - | ★ | ★ | ★ |
| Iltar 2021 | - | ★ | ★ | ★ | - | ★ | ★ | ★ | ★ |
| Ferraro 2022 | - | ★ | ★ | ★ | - | - | ★ | ★ | ★ |
| Chu 2015 | ★ | ★ | ★ | ★ | - | - | ★ | ★ | ★ |
| Lin 2020 | - | ★ | ★ | ★ | ★ | ★ | ★ | ★ | ★ |
| Armenian 2020 | ★ | ★ | ★ | ★ | - | - | ★ | ★ | ★ |
| Armenian 2019 | ★ | ★ | ★ | ★ | - | ★ | ★ | ★ | ★ |

Abbreviations: *: Patients from more than 1 hospital are considered as good representativeness; ★: yes; -: no

Table S4. Subgroup analysis of association between sarcopenia and overall survival for each variable.

| Variable | No. of Trials | OS, HR (95%CI) | *P* Value |
| --- | --- | --- | --- |
| Age |  |  | 0.480 |
| ＜60 | 9 | 1.66(1.40-1.97) |  |
| ≥ 60 | 18 | 1.49(1.15-1.93) |  |
| Male ratio |  |  | 0.630 |
| ＜55% | 12 | 1.51(1.08-2.11) |  |
| ≥ 55% | 15 | 1.66(1.39-1.97) |  |
| BMI |  |  | 0.620 |
| ＜25 | 11 | 1.65(1.23-2.22) |  |
| ≥25 | 10 | 1.49(1.13-1.97) |  |
| Follow-up period |  |  | 0.290 |
| ＜39 | 9 | 1.49(1.01-2.20) |  |
| ≥39 | 11 | 1.90(1.53-2.35) |  |
| IPI |  |  | 0.220 |
| ＜50% | 6 | 1.74(1.36-2.23) |  |
| ≥50% | 7 | 1.30(0.88-1.94) |  |
| SMI |  |  | 0.390 |
| ＜44 | 10 | 1.64(1.20-2.23) |  |
| ≥44 | 10 | 1.34(0.98-1.85) |  |
| Method to measure muscle |  |  | 0.060 |
| CT-L3-SMI | 21 | 1.48(1.23-1.79) |  |
| Other methods | 6 | 2.08(1.58-2.74) |  |
| Rate of sarcopenia |  |  | 0.620 |
| ＜50% | 13 | 1.73(1.42-2.09) |  |
| ≥50% | 12 | 1.57(1.15-2.16) |  |
| Sample size |  |  | 0.290 |
| ＜140 | 14 | 1.79(1.29-2.47) |  |
| ≥ 140 | 13 | 1.47(1.24-1.74) |  |
| Publication year |  |  | 0.550 |
| ＜2021 | 13 | 1.64(1.30-2.07) |  |
| ≥ 2021 | 14 | 1.49(1.15-1.95) |  |

Table S5. Subgroup analysis of association between sarcopenia and progression-free survival for each variable.

| Variable | No. of Trials | OS, HR (95%CI) | *P* Value |
| --- | --- | --- | --- |
| Age |  |  | 1.000 |
| ＜60 | 6 | 1.51(1.21-1.88) |  |
| ≥ 60 | 14 | 1.51(1.15-1.98) |  |
| Male ratio |  |  | 0.320 |
| ＜55% | 10 | 1.72(1.24-2.38) |  |
| ≥ 55% | 10 | 1.39(1.09-1.79) |  |
| BMI |  |  | 0.710 |
| ＜25 | 10 | 1.56(1.24-1.98) |  |
| ≥25 | 5 | 1.39(0.80-2.42) |  |
| Follow-up period |  |  | 0.720 |
| ＜39 | 7 | 1.59(1.10-2.29) |  |
| ≥39 | 9 | 1.72(1.37-2.15) |  |
| IPI |  |  | 0.460 |
| ＜50% | 5 | 1.63(1.22-2.19) |  |
| ≥50% | 6 | 1.35(0.90-2.03) |  |
| SMI |  |  | 0.150 |
| ＜43 | 7 | 1.84(1.20-2.82) |  |
| ≥43 | 8 | 1.22(0.86-1.74) |  |
| Method to measure muscle |  |  | 0.860 |
| CT-L3-SMI | 16 | 1.53(1.17-2.00) |  |
| Other methods | 4 | 1.48(1.19-1.85) |  |
| Rate of sarcopenia |  |  | 0.610 |
| ＜44% | 9 | 1.67(1.30-2.13) |  |
| ≥44% | 10 | 1.51(1.15-1.99) |  |
| Sample size |  |  | 0.170 |
| ＜125 | 10 | 1.82(1.25-2.65) |  |
| ≥ 125 | 10 | 1.35(1.09-1.68) |  |
| Publication year |  |  | 0.400 |
| ＜2021 | 9 | 1.66(1.22-2.25) |  |
| ≥ 2021 | 11 | 1.40(1.08-1.81) |  |

Table S6. Subgroup analysis of association between sarcopenia and complete response for each variable.

|  |  | No. of Patients | |  |  |
| --- | --- | --- | --- | --- | --- |
| Variable | No. of Trials | With CR | Total | CR, OR (95%CI) | *P* Value |
| Age |  |  |  |  | 0.320 |
| ＜60 | 4 | 267 | 451 | 0.47(0.32-0.70) |  |
| ≥ 60 | 5 | 421 | 651 | 0.62(0.43-0.90) |  |
| **Male ratio** |  |  |  |  | **0.020** |
| ＜55% | 4 | 281 | 470 | 0.82(0.52-1.28) |  |
| ≥ 55% | 5 | 407 | 632 | 0.42(0.30-0.60) |  |
| **BMI** |  |  |  |  | **0.020** |
| ＜25 | 3 | 223 | 374 | 0.92(0.54-1.57) |  |
| ≥25 | 4 | 345 | 542 | 0.42(0.29-0.62) |  |
| Follow-up period |  |  |  |  | 0.100 |
| ＜50 | 3 | 187 | 274 | 0.52(0.30-0.90) |  |
| ≥50 | 3 | 186 | 339 | 0.99(0.58-1.68) |  |
| IPI |  |  |  |  | 0.890 |
| ＜50% | 3 | 276 | 429 | 0.57(0.29-1.09) |  |
| ≥50% | 3 | 225 | 399 | 0.61(0.27-1.41) |  |
| SMI |  |  |  |  | 0.160 |
| ＜47 | 4 | 285 | 464 | 0.76(0.47-1.24) |  |
| ≥47 | 3 | 271 | 422 | 0.47(0.30-0.75) |  |
| Method to measure muscle |  |  |  |  | 0.750 |
| CT-L3-SMI | 6 | 427 | 722 | 0.53(0.37-0.75) |  |
| Other methods | 3 | 261 | 480 | 0.57(0.37-0.90) |  |
| Rate of sarcopenia |  |  |  |  | 0.520 |
| ＜50% | 3 | 259 | 450 | 0.68(0.43-1.10) |  |
| ≥50% | 4 | 198 | 383 | 0.55(0.34-0.88) |  |
| Sample size |  |  |  |  | 0.330 |
| ＜145 | 5 | 291 | 447 | 0.47(0.31-0.71) |  |
| ≥145 | 4 | 497 | 651 | 0.61(0.43-0.88) |  |

**Figure S1**. Funnel plots assessing publication bias. (A) OS; (B) PFS; (C) CR.


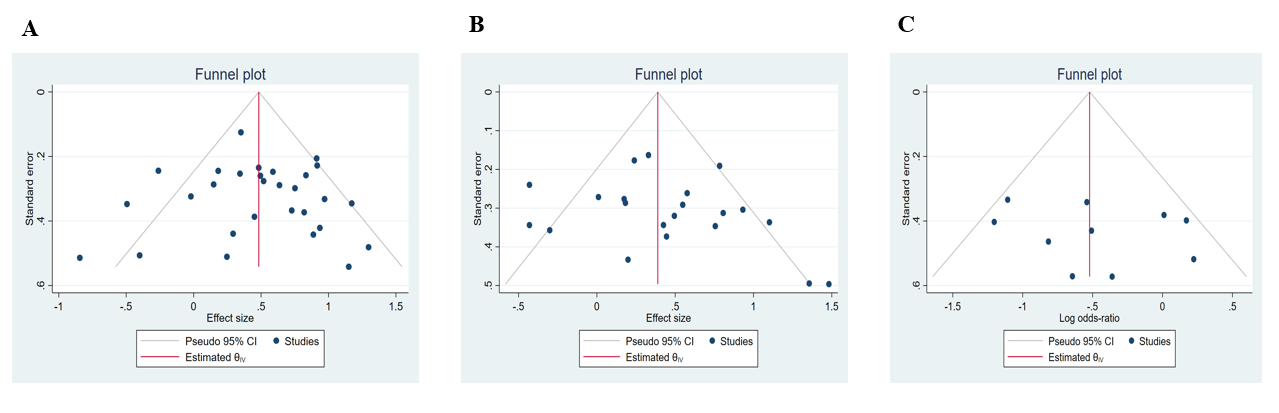


**Figure S2**. Sensitivity analysis for prevalence of sarcopenia prior to treatment in patients with hematological malignancies.

**
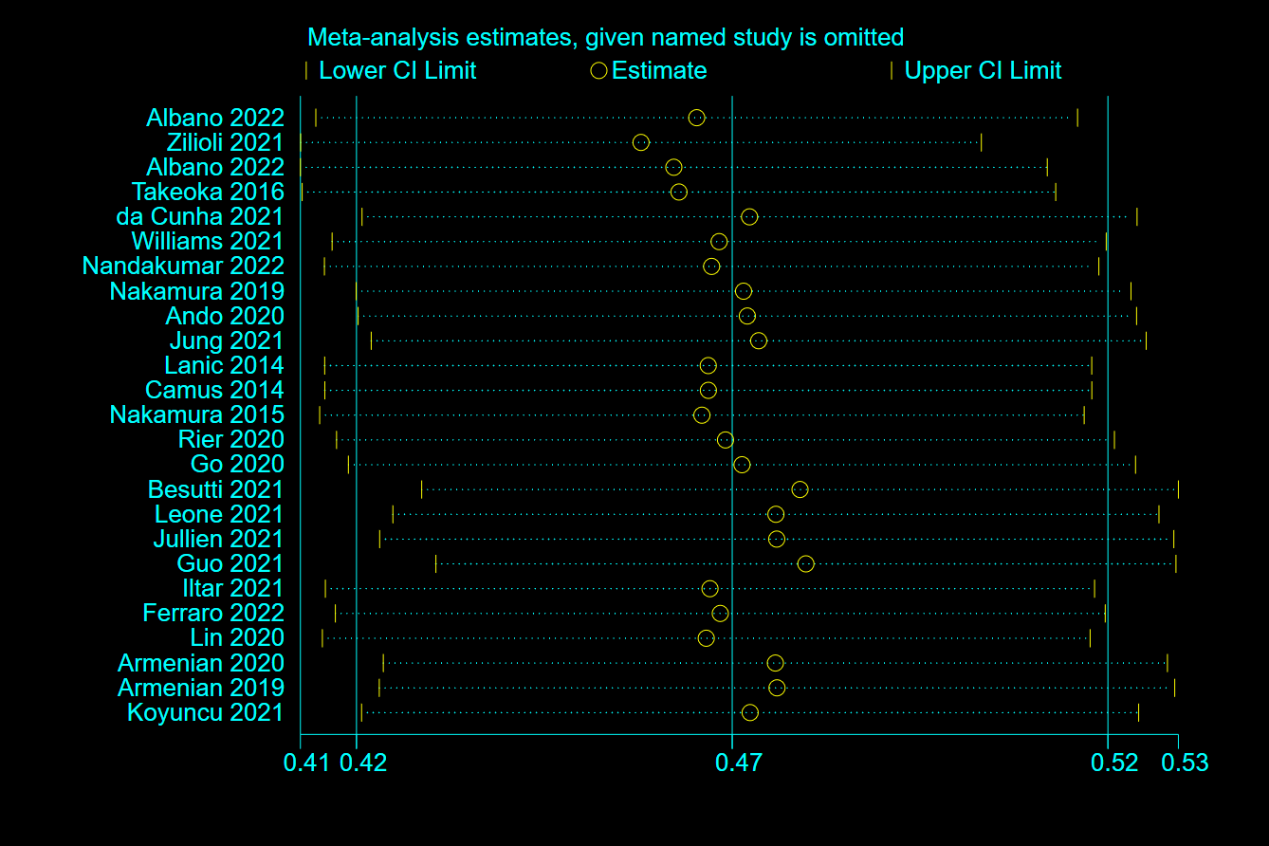
**

**Figure S3**. Sensitivity analysis for overall survival.

**
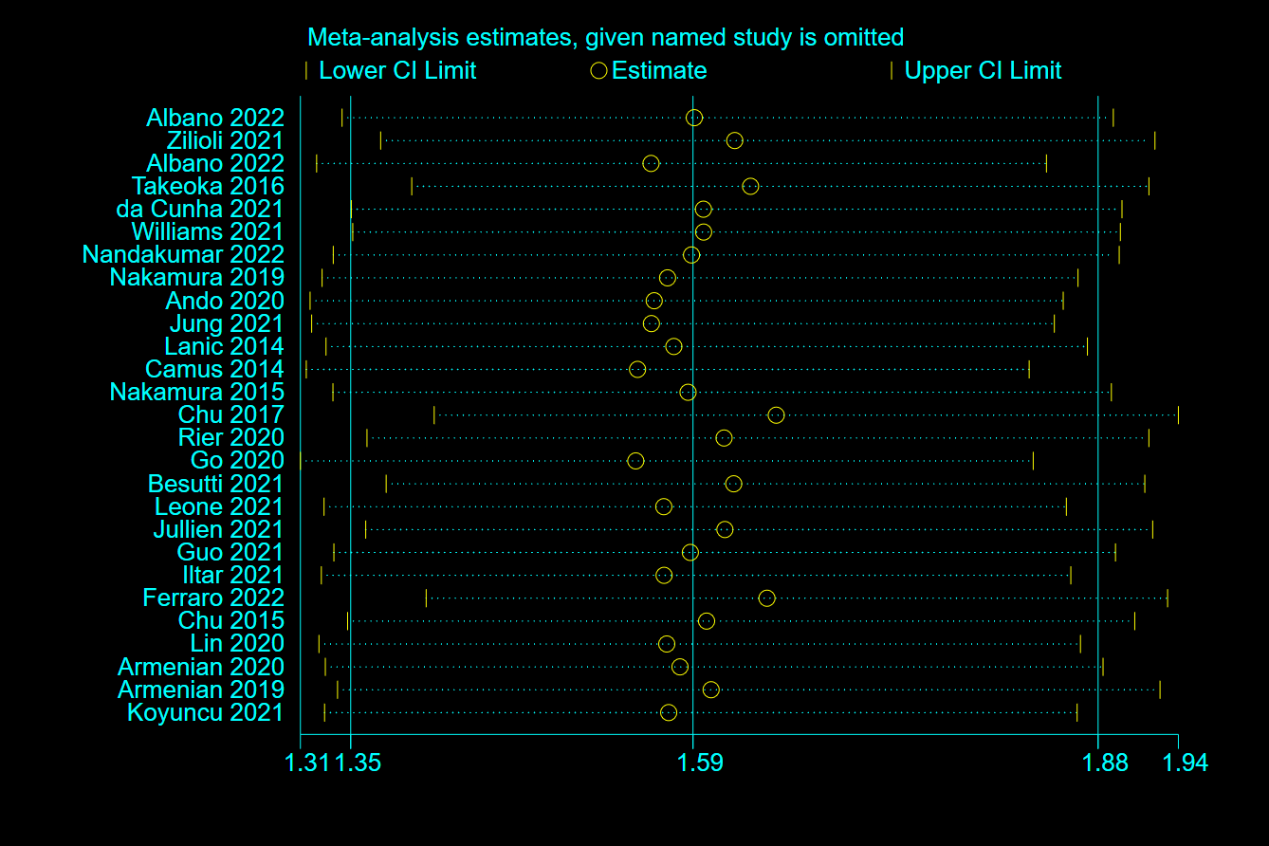
**

**Figure S4**. Sensitivity analysis for progression-free survival.

**
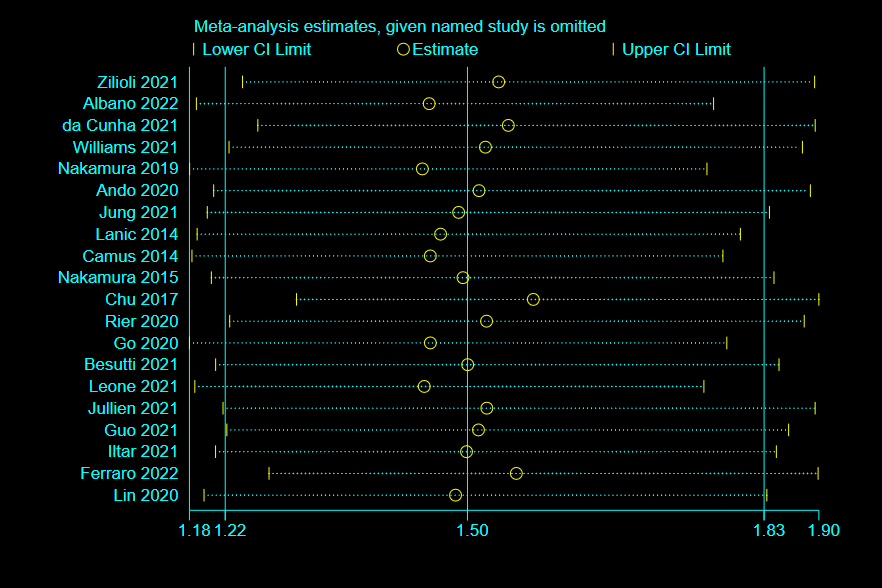
**

**Figure S5**. Sensitivity analysis for complete response.

**
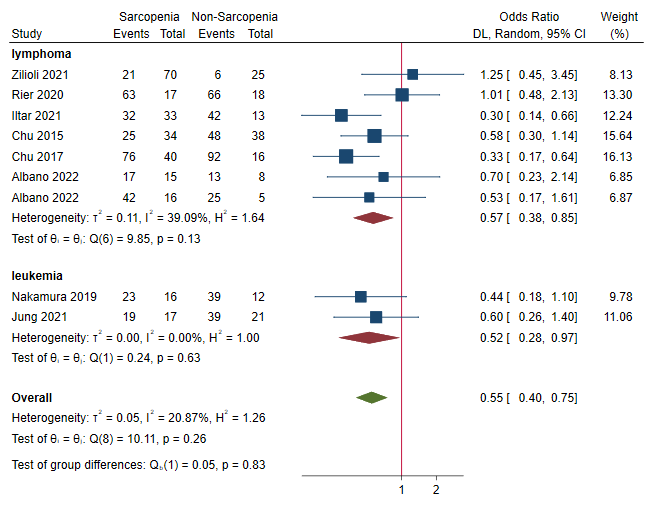
**

1. Go SI, Park S, Kang MH, Kim HG, Kim HR, Lee GW. Clinical impact of prognostic nutritional index in diffuse large B cell lymphoma. *Ann Hematol* 2019; **98**(2): 401-11.

2. Go SI, Park MJ, Song HN, et al. A comparison of pectoralis versus lumbar skeletal muscle indices for defining sarcopenia in diffuse large B-cell lymphoma - two are better than one. *Oncotarget* 2017; **8**(29): 47007-19.

3. Go SI, Park MJ, Song HN, et al. Prognostic impact of sarcopenia in patients with diffuse large B-cell lymphoma treated with rituximab plus cyclophosphamide, doxorubicin, vincristine, and prednisone. *Journal of cachexia, sarcopenia and muscle* 2016; **7**(5): 567-76.

4. da Cunha Júnior AD, Silveira MN, Takahashi MES, et al. Visceral adipose tissue glucose uptake is linked to prognosis in multiple myeloma patients: An exploratory study. *Clinical nutrition (Edinburgh, Scotland)* 2021; **40**(6): 4075-84.

5. Xiao DY, Luo S, O'Brian K, et al. Impact of sarcopenia on treatment tolerance in United States veterans with diffuse large B-cell lymphoma treated with CHOP-based chemotherapy. *Am J Hematol* 2016; **91**(10): 1002-7.

6. Tagliafico AS, Rossi F, Bignotti B, et al. CT-derived relationship between low relative muscle mass and bone damage in patients with multiple myeloma undergoing stem cells transplantation. *The British journal of radiology* 2022; **95**(1132): 20210923.

7. Jabbour J, Manana B, Zahreddine A, et al. Sarcopenic obesity derived from PET/CT predicts mortality in lymphoma patients undergoing hematopoietic stem cell transplantation. *Current research in translational medicine* 2019; **67**(3): 93-9.

8. Umit EG, Korkmaz U, Baysal M, et al. Evaluation of Sarcopenia with F-18 FDG PET/CT and relation with disease outcomes in patients with multiple myeloma. *European journal of cancer care* 2020; **29**(6): e13318.

9. Bonm A, Menghini A, Graber J. Sarcopenia as measured by temporalis muscle width is a predictor of survival in primary CNS lymphoma. *Neuro-Oncology* 2021; **23**(SUPPL 6): vi145.

10. Leone R, Sferruzza G, Calimeri T, et al. Quantitative muscle mass biomarkers are independent prognosis factors in primary central nervous system lymphoma: The role of l3-skeletal muscle index and temporal muscle thickness. *Neuro-Oncology* 2021; **23**(SUPPL 2): ii48.

11. Mishra A, Thapa R, Bigam K, et al. CT-Defined Fat Index Is a Prognostic Factor of Chronic Graft-Versus-Host Disease Outcomes in Adult Allogeneic Transplant Recipients. *Biology of Blood and Marrow Transplantation* 2020; **26**(3): S183-S4.

12. Go SI, Park MJ, Park S, et al. Cachexia index as a potential biomarker for cancer cachexia and a prognostic indicator in diffuse large B-cell lymphoma. *Journal of cachexia, sarcopenia and muscle* 2021; **12**(6): 2211-9.

13. Jaswal S, Sanders V, Pullarkat P, et al. Metabolic Biomarkers Assessed with PET/CT Predict Sex-Specific Longitudinal Outcomes in Patients with Diffuse Large B-Cell Lymphoma. *Cancers* 2022; **14**(12).

14. Kondo S, Kagawa K, Saito T, et al. Allogeneic haematopoietic stem cell transplantation-clinical outcomes: impact of leg muscle strength. *BMJ supportive & palliative care* 2021.

15. Koyuncu MB, Guler E, Koseci T, et al. Integration of low muscle mass into the IPS system and its prognostic significance in patients with Hodgkin's lymphoma. *Biomarkers in medicine* 2022; **16**(2): 57-67.

16. Lucijanic M, Huzjan Korunic R, Sedinic M, et al. Baseline and progressive adipopenia in newly diagnosed patients with diffuse large B-cell lymphoma with unfavorable features are associated with worse clinical outcomes. *Leuk Lymphoma* 2022; **63**(7): 1556-65.

17. Lucijanic M, Huzjan Korunic R, Sedinic M, Kusec R, Pejsa V. More Pronounced Muscle Loss During Immunochemotherapy is Associated with Worse Clinical Outcomes in Newly Diagnosed Patients with Diffuse Large B-Cell Lymphoma with Unfavorable Features. *Therapeutics and clinical risk management* 2021; **17**: 1037-44.

18. Nagata A, Otsuka Y, Konuma R, et al. Weight-adjusted urinary creatinine excretion predicts transplant outcomes in adult patients with acute myeloid leukemia in complete remission. *Leuk Lymphoma* 2022; **63**(13): 3117-27.

19. Nagayama T, Fujiwara SI, Kikuchi T, et al. Impact of muscle mass loss assessed by computed tomography on the outcome of allogeneic stem cell transplantation. *Leuk Lymphoma* 2022; **63**(7): 1694-700.

20. Shen H, Zhao Y, Shi Y, et al. The diagnostic and prognostic value of MRI in central nervous system involvement of acute myeloid leukemia: a retrospective cohort of 84 patients. *Hematology (United Kingdom)* 2020; **25**(1): 258-63.

21. Yilmaz M, Atilla FD, Sahin F, Saydam G. The effect of malnutrition on mortality in hospitalized patients with hematologic malignancy. *Supportive Care in Cancer* 2020; **28**(3): 1441-8.

22. Amini B, Nakache YN, Nardo L, et al. Marrow uptake on FDG PET/CT is associated with progression from smoldering to symptomatic multiple myeloma. *Skeletal Radiol* 2021; **50**(1): 79-85.

23. Hinnerichs M, Ferraro V, Zeremski V, et al. Prognostic Impact of Quality and Distribution of Adipose Tissue in Patients With Primary Central Nervous System Lymphoma. *In vivo (Athens, Greece)* 2022; **36**(6): 2828-34.

24. Menghini A, Bonm A, Graber J. Sarcopenia Measured by Temporalis Muscle Thickness Independently Predicts Early Relapse and Short Survival in Primary CNS Lymphoma. *Neurology* 2022; **98**(18 SUPPL).

25. Lucijanic M, Huzjan Korunic R, Ivic M, et al. Psoas muscle index at the time of diagnosis might reflect the prognosis of classical Hodgkin's lymphoma patients. *Wiener klinische Wochenschrift* 2022; **134**(1-2): 80-2.

26. Sun Q, Cui J, Liu W, Li J, Hong M, Qian S. The Prognostic Value of Sarcopenia in Acute Myeloid Leukemia Patients and the Development and Validation of a Novel Nomogram for Predicting Survival. *Front Oncol* 2022; **12**: 828939.

27. Furtner J, Nenning KH, Roetzer T, et al. Evaluation of the Temporal Muscle Thickness as an Independent Prognostic Biomarker in Patients with Primary Central Nervous System Lymphoma. *Cancers* 2021; **13**(3).
